# Thickness-Dependent Band Gap Modification in BaBiO_3_

**DOI:** 10.3390/nano11040882

**Published:** 2021-03-30

**Authors:** Rosa Luca Bouwmeester, Alexander Brinkman, Kai Sotthewes

**Affiliations:** MESA+ Institute for Nanotechnology, University of Twente, 7500 AE Enschede, The Netherlands; a.brinkman@utwente.nl (A.B.); k.sotthewes@utwente.nl (K.S.)

**Keywords:** BaBiO_3_, scanning tunneling miscroscopy, spectroscopy, pulsed laser deposition, perovskite, complex oxide, band gap, surface reconstruction

## Abstract

The material BaBiO${}_{3}$ is known for its insulating character. However, for thin films, in the ultra-thin limit, metallicity is expected because the oxygen octahedra breathing mode will be suppressed as reported recently. Here, we confirm the influence of the oxygen breathing mode on the size of the band gap. The electronic properties of a BaBiO${}_{3}$ thickness series are studied using in-situ scanning tunneling microscopy. We observe a wide-gap ($E_{G}$ > 1.2 V) to small-gap ($E_{G}$ ≈ 0.07 eV) semiconductor transition as a function of a decreasing BaBiO${}_{3}$ film thickness. However, even for an ultra-thin BaBiO${}_{3}$ film, no metallic state is present. The dependence of the band gap size is found to be coinciding with the intensity of the Raman response of the breathing phonon mode as a function of thickness.

## 1. Introduction

The material system BaBiO${}_{3}$ (BBO) received a lot of attention since it was first fabricated in 1963 [1] and is currently applied in various fields as in photoelectrochemical water splitting processes [2] and as an absorber in solar cells [3]. In a simple ionic picture, a Bi${}^{4+}$ ion is expected to be present. Its half-filled 6*s* shell would make BBO metallic [4,5,6,7]. Contrary, experimental results showed an insulating character [7,8,9] with an optically observed bulk bandgap of 2 eV [10,11,12], raising the question of what mechanism is responsible for its insulating behavior. When more studies were performed, it was discovered that BBO became superconducting upon hole doping [4,5,6,12,13,14]. With optimum lead or potassium doping, critical temperatures of 13 K [6] and 30 K [14], respectively, were achieved. It is still an open question why BaPb${}_{1-x}$Bi${}_{x}$O${}_{3}$ and Ba${}_{1-x}$K${}_{x}$BiO${}_{3}$ become superconducting. A possible explanation might be related to the mechanism causing the unexpected insulating behavior in BBO. In this work, the electronic properties are studied to shine a new light on the mechanism responsible for the insulating character.

At first, a charge disproportionation, with alternating 3+ and 5+ valence states of the Bi atom, was given as the origin for the insulating behavior [7,10,15,16]. Subsequently, others claimed the oxygen-breathing mode [8,11,17,18]—where the oxygen octahedra contract and expand—to be responsible for the bandgap formation in BBO. In a more recent view, a strong hybridization between the Bi 6*s* and O 2*p* states creates a bond disproportionation [19,20,21]. Here, all bismuth ions have an oxidation state of 3+ [21,22], but different local environments. A hole pair sits on the contracted oxygen octahedra [21], explaining the experimentally observed two different Bi–O bond lengths [7,10,15]. In experimental work, the presence of an oxygen 2*p* hole density in the ground state was observed for BBO single crystals [23] and a Bi core level analysis of BBO thin films revealed the presence of solely Bi${}^{3+}$ ions [24], both agreeing with the theoretically proposed bond disproportionated picture.

In both the charge disproportionated and bond disproportionated picture, the oxygen octahedra breathing mode is present. The appearance of this breathing mode combined with octahedra tilting distortions cause a phase transition from a cubic to a monoclinic structure [16]. When considering the thickness of the BBO films as a degree of freedom, it was found that for films thinner than 9 unit cells (u.c.) the structure transforms from a tetragonal to a cubic phase [25], hinting towards suppression of the oxygen-breathing mode. Theory shows a closing of the bandgap when the oxygen octahedra breathing mode is excluded and a cubic BBO crystal structure is considered [21]. Nevertheless, no electrical properties were determined for any of these ultra-thin BBO films in order to confirm the presence or absence of a thickness-dependent insulator-to-metal transition.

Furthermore, with a Raman spectroscopy experiment on the same series of BBO films, suppression of the breathing phonon intensity was observed as a function of thickness. Repeating the experiment for BBO films deposited on a double buffer layer template of BaZrO${}_{3}$ and BaCeO${}_{3}$, suppression of the breathing phonon intensity was not observed until 6 u.c. [26]. When BBO films were deposited directly on an Nb-doped SrTiO${}_{3}$(001) substrate, the breathing phonon intensity was present till a thickness of 7 u.c. [27]. However, it was concluded that the loss of response intensity was caused by a reconstruction layer at the interface (see refs. [28,29]), rather than by suppression of the oxygen-breathing mode.

Here, we study the electronic properties of a BBO thickness series by in-situ scanning tunneling spectroscopy (STS) experiments. We found that the size of the bandgap ($E_{G}$) depends on the thickness of the BBO film and shrinks from $E_{G}$ > 1.2 V for a 16-unit-cell-thick film to $E_{G}$ ≈ 0.07 eV for a film with a thickness of 3 unit cells. A c(4 × 2) surface reconstruction confirms the presence of a perovskite structure.

## 2. Materials and Methods

BBO films are fabricated with thicknesses ($d_{\mathrm{BBO}}$) of 4, 10 and 16 u.c. on TiO${}_{2}$-terminated, 0.5 wt% Nb-doped SrTiO${}_{3}$(001) substrates (Nb:STO) from CrysTec GmbH (Berlin, Germany). In the Supplementary materials, see Figure S1, an atomic force microscopy (AFM) image and corresponding height profile of the substrate are presented. The films were fabricated by pulsed laser deposition (PLD) using a stoichiometric house-made BaBiO${}_{3}$ target (purity 99.99%). The growth conditions of the BBO films were the same as reported in earlier work [29]. In the Supplementary Materials, more details on sample preparation are provided together with the reflection high-energy electron diffraction (RHEED) patterns and intensity curves, see Figure S2. Furthermore, AFM images of the BBO films are shown in Figures S3 and S4a–d for the thicknesses 4, 10 and 16 u.c., respectively.

Subsequently, the BBO samples were transferred in-situ to an Omicron (Taunusstein, Germany) Nanoprobe scanning tunneling microscope (STM), with a base pressure of 1 × 10${}^{-10}$ mbar using chemically-etched tungsten tips. The measurements were acquired at room temperature. All voltages refer to the tip bias voltage with respect to the sample. The $\frac{dI}{dV}\left(V\right)$ curves were recorded using a lock-in amplifier (*f* = 1763 Hz, $V_{\mathrm{AC}}$ = 30 mV). In addition, I(V) curves were recorded and used for calibration of the $\frac{dI}{dV}\left(V\right)$ curves. In order to compare the band gap obtained from the different samples, the curves are normalized with respect to each other. By plotting the corrected I(V) spectra on a semi-logarithmic scale, the bandgap is determined by taking the average voltage separation between the conduction and valence band current onsets at the lowest detectable current (detection limit approximately 500 fA) [30,31,32], as explained in more detail in the Supplementary Materials and presented in Figure S7.

## 3. Results

**Figure 1:**
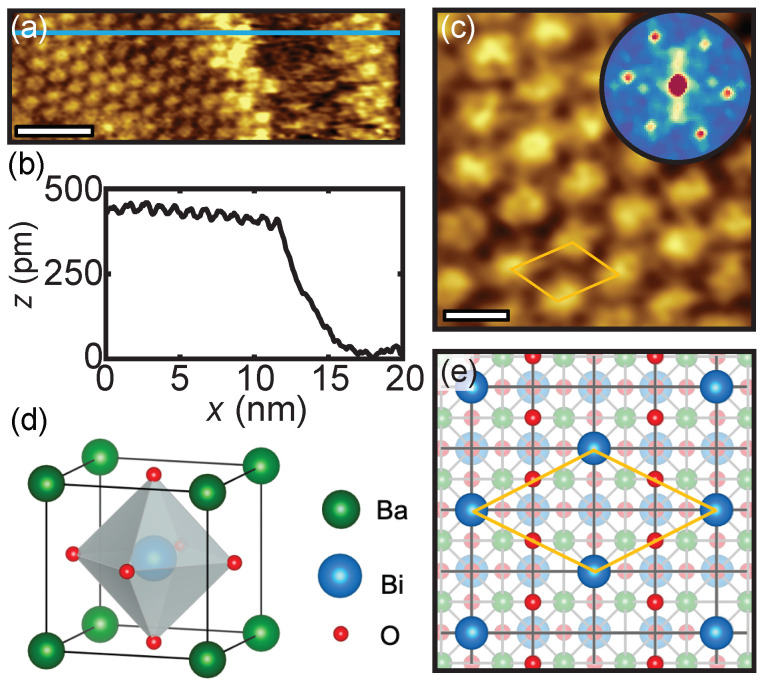
(**a**) Topography image (20 × 7 nm, scale bar is 4 nm) of the 10-unit-cell-thick BaBiO${}_{3}$ (BBO) film on Nb:STO showing a stepped surface. (**b**) Cross-sectional height profile along the corresponding light blue line segment in (**a**). The step height is approximately 4.5 Å. (**c**) Zoomed image (5 × 5 nm, scale bar 1 nm) showing the c(4 × 2) surface reconstruction, the diamond-shaped orange lines indicate the unit cell. Inset: The corresponding Fast Fourier transform (FFT) showing a threefold symmetry with a periodicity of 1 nm. (**d**) Schematic of the cubic perovskite BBO unit cell. The gray-shaded area represents an oxygen octaheder. (**e**) A top view of the c(4 × 2) surface reconstruction. The diamond-shaped orange lines show the unit cell (corresponding to the orange diamond shape in (**c**)).

Figure 1a shows the surface of the 10-unit-cell-thick BBO film on Nb:STO. The corresponding height profile across the surface is presented in Figure 1b and reveals a step height of approximately 4.5 Å. This is consistent with a single unit cell of BBO (*a* = 4.35 Å) [29]. Figure 1c shows a zoomed image of the BBO surface. The observed pattern of atoms corresponds to a c(4 × 2) surface reconstruction, the diamond-shaped orange lines indicate its unit cell. The same surface reconstruction is also found for the 4-unit-cell-thick BBO film, shown in Figure S5 of the Supplementary Materials. In Figure 1e the surface reconstruction on the BBO surface is schematically depicted, the orange diamond-shaped lines indicate the unit cell and correspond to the orange lines in Figure 1c.

Erdman et al. [33] concluded for the c(4 × 2) reconstruction on a STO(001) surface that a stoichiometric TiO${}_{2}$ overlayer was present, consisting of TiO${}_{5}$ edge-sharing polyhedra. Such an overlayer is formed to stabilize the truncated, corner-sharing octahedra in the surface layer of a TiO${}_{2}$-terminated STO(001) underneath. Therefore, we propose, in analogy with the studies on Nb:STO(001) and STO(001), that a BiO${}_{2}$ overlayer is present on the surface of BBO, hosting BiO${}_{5}$ edge-sharing polyhedra.

The Fast Fourier transform (FFT), shown in the inset of Figure 1c, has a threefold symmetry with a periodicity of 1 nm. Note that the underlying crystal structure is still a fourfold symmetric cubic perovskite structure. The lattice constant corresponding to a periodicty of 1 nm is 4.35 Å ($\sqrt{5}\;\times$ 4.35 Å = 1 nm), which is in agreement with the BBO lattice constant. The surface reconstruction on the 4-unit-cell-thick BBO film also has a periodicity of 1 nm, as presented in the inset of Figure S5 in the Supplementary Materials. Previous studies on Nb:STO(001) and STO(001) surfaces revealed the same type of reconstruction, however, with a periodicity of 0.88 nm—corresponding to a bulk lattice constant of 3.9 Å [33,34,35] matching STO (*a* = 3.905 Å) [36].

The observation of BBO thin films with a relaxed lattice constant is in good agreement with our previous study [29], where we show—by means of a scanning transmission electron microscopy (STEM)—that a lattice mismatch of 12% between the STO substrate and BBO film is accommodated by the formation of an interfacial layer. This was confirmed by Jin et al. [37], where an interfacial layer with a fluorite structure and similar thickness is observed at the STO/BBO interface. The interfacial layer decouples the BBO film from the substrate. The bottommost part is still strained to the STO substrate, but the subsequent layer is already fully decoupled and relieves all strain in a dislocation every ninth unit cell [29]. Therefore, the substrate does not influence the BBO film.

The BBO film is able to continue its growth without any effects of strain in a perovskite structure [29,37]. Anti-phase boundaries, where a step of half a unit cell is present, are observed within the BBO film in various STEM studies [28,29,37]. However, this boundary does not disturb the quality of the film. The suggested suppression of the oxygen-breathing mode as a function of thickness has not yet been observed by STEM. The structural transition from tetragonal to cubic, as previously observed [25], is too small to be detected by STEM since it occurs on the picometer range.

The presence of the surface reconstruction, with an identical periodicity for the 4- and 10-unit-cell-thick BBO films, proves that the underlying structure in both cases is still the perovskite structure with the relaxed lattice constant of bulk BBO. The subsequently presented spatially-resolved STS measurements are, therefore, observing the effect of thickness and not of substrate-induced strain nor defects.

**Figure 2:**
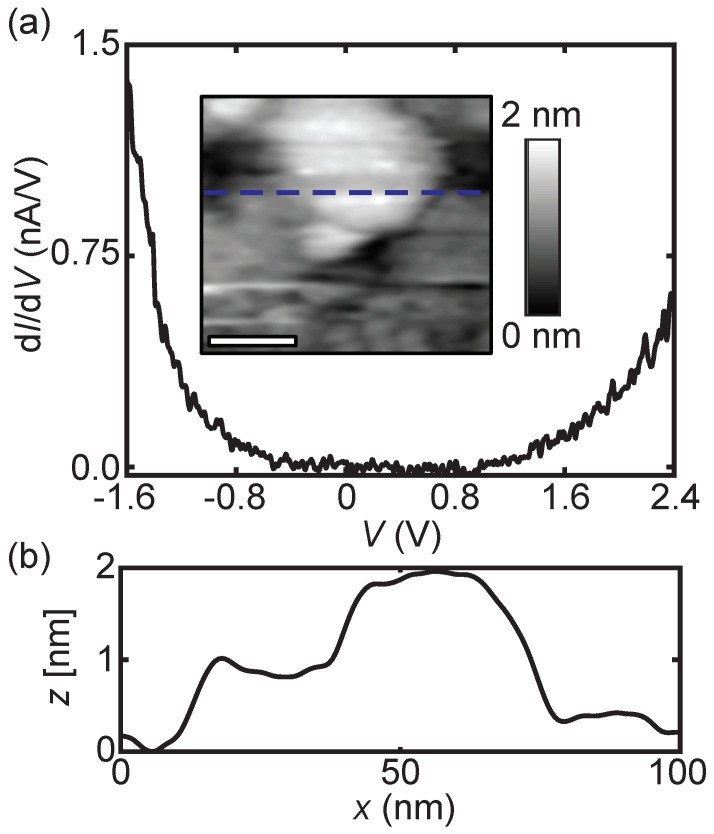
Differential conductivity spectra ($\frac{dI}{dV}\left(V\right)$) on the 16-unit-cell-thick BBO film on Nb:STO(001). A band gap of approximately 1.2 ± 0.3 eV is observed. Inset: Topography image (100 × 100 nm, scale bar 30 nm). The tunneling parameters are 500 pA and −1.8 V. (**b**) Cross-sectional height profile of the corresponding blue dashed line in the inset of (**a**).

STS was first used to determine the electronic properties of the 16-unit-cell-thick BBO film. In Figure 2a, the differential conductance curves ($\frac{dI}{dV}\left(V\right)$) represent the local density of states (LDOS). A semiconducting characteristic with a bandgap ($E_{G}$) of 1.2 ± 0.3 eV is observed. From the cross-section height profile (see Figure 2b) variations of 2 nm are observed, which have no clear spatial-dependent influence on the electronic profile across the surface. The bandgap is asymmetric with the valence band located closer to the Fermi level ($E_{F}$).

**Figure 3:**
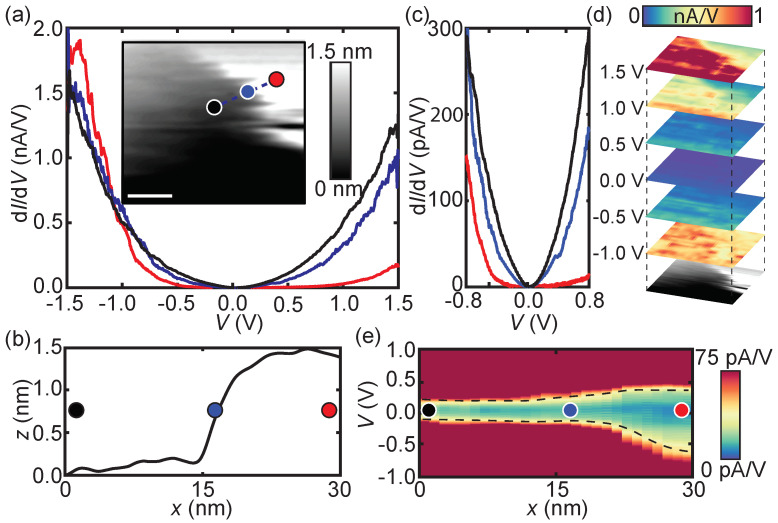
(**a**) Differential conductivity spectra ($\frac{dI}{dV}\left(V\right)$) on the 10-unit-cell-thick BBO film on Nb:STO, the curve colors correspond to the positions (colored dots) marked in the inset. A wide bandgap (red) to small bandgap (black) transition is found depending on the thickness of the film. Inset: Topography image (100 × 100 nm, scale bar 25 nm) with spectrum locations marked by the colored dots. The tunneling parameters are 700 pA and −1.5 V. (**b**) Cross-sectional height profile, from the corresponding blue dashed line in the inset of (**a**), showing a height difference of 1.5 nm between the higher and lower region. (**c**) Zoom-in of the spectroscopy data of (**a**) revealing a decrease of the bandgap when moving from the higher to the lower region. (**d**) d*I*/d*V* maps at different bias voltage set points. The lateral position is aligned with the topography image at the bottom, which is the same as the inset in (**a**). An increased local density of states (LDOS) at the lower region of the sample is clearly visible for *V* > 1 *V*. (**e**) $\frac{dI}{dV}\left(V\right)$ cross-section recorded across a transition region (colored dots correspond to the dots in the inset of (**a**)). A strong decrease of the bandgap is observed when going from the higher (red dot) to the lower (black dot) region. The black dashed line is a guide to the eye.

In the case of the 10-unit-cell-thick BBO film, the LDOS varies spatially. Figure 3a shows the LDOS on various locations on the sample, the colored dots on the topography image in the inset correspond to colors of the differential conductance spectra. On the thicker part of the sample, represented by the red curve in Figure 3a, semiconducting behavior is observed—similar to the profile on the 16-unit-cell-thick BBO film in Figure 2a. However, the size of the band gap is reduced to 0.7 ± 0.2 eV.

From the cross-section height profile, see Figure 3b, it is clear that the lower region (black dot) is located 1.4 nm (≈3 u.c.) lower with respect to the higher region (red dot). Note that the exact thickness of the BBO film is unknown and therefore only relative thicknesses are given. The $\frac{dI}{dV}\left(V\right)$ spectra taken at the lower region of the sample, the black curve in Figure 3a, reveal a significant reduction of $E_{G}$. A closer look at the Fermi energy, depicted in Figure 3c, shows this even more clearly. The bandgap reduces to approximately 0.10 ± 0.03 eV.

The simultaneously obtained differential conductance (d*I*/d*V*) maps, presented in Figure 3d, depict the LDOS for the thickness regions at different bias voltages. A clear correlation is observed between the topography, bottommost image, and the LDOS. At non-zero bias voltages, the LDOS is much higher at the lower region of the sample compared to the higher region. At zero bias voltage, all the contrast is lost and the regions can no longer be distinguished, excluding the presence of a metallic state.

In between the two height regions, a transition region is present, indicated by the blue dot in the inset of Figure 3a and by the blue curve in Figure 3a,c. The size of the bandgap depends heavily on the exact location of the sample, this is better visualized in Figure 3e. The measured $\frac{dI}{dV}\left(V\right)$ curves are plotted as a function of the distance, the colored dots correspond to the dots in the inset of Figure 3a. A continuous but steep transition in the bandgap size is found between the two regions.

**Figure 4:**
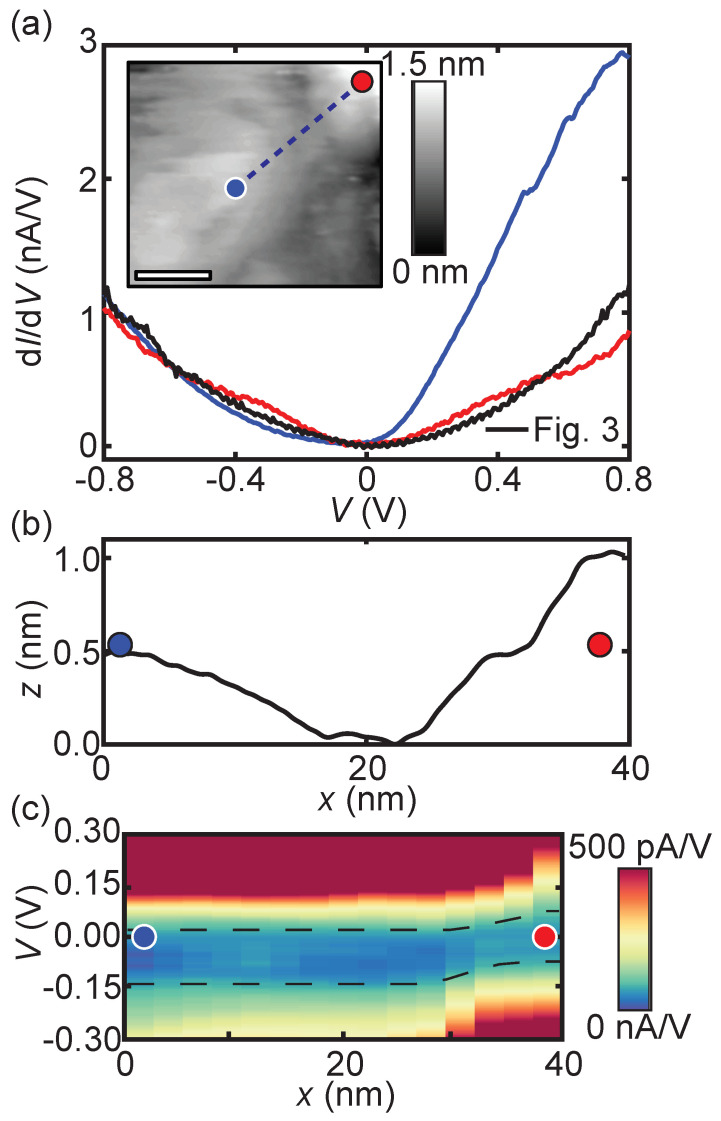
(**a**) $\frac{dI}{dV}\left(V\right)$ spectra on the 4-unit-cell-thick BBO film on Nb:STO according to the positions (blue and red dot) marked in the inset. The black curve is taken from Figure 3c (the lower region) and rescaled based on the tunneling parameters. Inset: Topography image (80 × 80 nm, scale bar 20 nm) with the spectrum locations marked. The tunneling parameters are 400 pA and −1 V. (**b**) Cross-sectional height profile from corresponding blue dashed line in (a), a height variation of maximum 1 nm is observed. (**c**) $\frac{dI}{dV}\left(V\right)$ cross-section recorded across a transition region (blue dashed line in the inset of (**a**)).

To further confirm the observed bandgap reduction, the 4-unit-cell-thick BBO film is studied. Figure 4a shows the location-dependent differential conductance. The LDOS measured on the higher region of the 4 u.c. BBO film (red curve in Figure 4a) has the same characteristics as the spectrum measured on the lower region of the 10 u.c. BBO film (black curve in Figure 3a,c), including a similar bandgap of 0.10 ± 0.05 eV. For better visualization, the scaled differential conductance spectrum of Figure 3c (black curve) is also plotted in Figure 4a. The d*I*/d*V* maps at different bias voltage set points for the 4-unit-cell-thick film are presented in the Supplementary Materials in Figure S6.

On the region located 1 u.c. lower, see the height profile in Figure 4b, the LDOS is altered again (blue curve in Figure 4a). In addition to a small decrease in the size of the bandgap (i.e., 0.07 ± 0.04 eV), the bandgap is no longer positioned symmetrically relative to the Fermi energy. A small shift towards negative voltages is observed (i.e., the conductance band is located closer to $E_{F}$), which is most likely caused by band bending. The contact potential difference between Nb:STO and BBO results in an accumulation layer of electrons in the BBO film, bending the conduction band towards the Fermi energy. Figure 4c shows the $\frac{dI}{dV}\left(V\right)$ as a function of the distance. The shift of the bandgap towards more negative voltages at the lower region (blue dot) is clearly observed.

From the BBO thin film thickness series, we observed the following: (1) a reduction of the bandgap as a function of decreasing thickness, (2) the absence of a metallic state and (3) a monotonous but steep transition in the bandgap size between the different thickness regions. Although an insulator-to-metal transition was predicted [25], a clear wide-gap ($E_{G}$ > 1.2 eV) to small-gap ($E_{G}$ ≈ 0.07 eV) semiconductor transition is observed as a function of $d_{\mathrm{BBO}}$.

**Figure 5:**
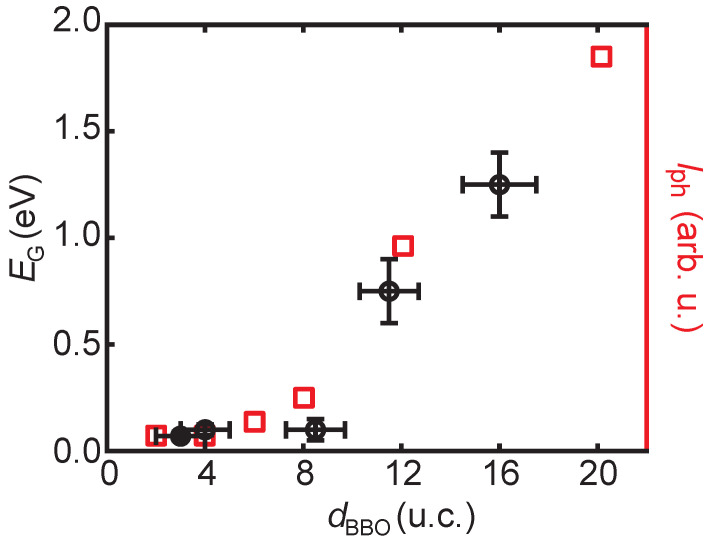
The band gap (left axis, black circles) and the relative intensity of the Raman response of the breathing phonon mode ($I_{\mathrm{ph}}$) (right axis, red squares, taken from ref. [27]) as a function of the BBO film thickness ($d_{\mathrm{BBO}}$). A similar trend is found for $E_{G}$ and $I_{\mathrm{ph}}$. The *x*-axis error bar is the root-mean-square (RMS) roughness extracted from atomic force microscopy images (see Supplementary Materials, Figures S3 and S4).

In Figure 5, the determined band gap sizes (see Figure S7 of the Supplementary Materials) are plotted as a function of thickness. The uncertainty in thickness for the BBO films is determined from AFM images, presented in the Supplementary Materials, see Figures S3 and S4. When measurements are repeatedly performed on BBO films, similar *I*(V) curves are obtained, as shown in Figure S8 of the Supplementary Materials. The transition from a wide-gap to a small-gap semiconductor is continuous and gradual.

Recent density functional theory (DFT) calculations in combination with a tight-binding (TB) model [21] revealed the influence of the oxygen-breathing mode on the band structure of BBO. If the oxygen-breathing mode is absent, a metallic band structure is predicted, while a bandgap forms when the oxygen-breathing mode is present. Implying that a semiconductor-metal transition occurs when the oxygen-breathing mode is suppressed, as we observe as a function of thickness. No metallicity is observed, even for the 4-unit-cell-thick BBO film, implying that the oxygen-breathing mode is not fully suppressed.

On the right axis of Figure 5, the intensity of the Raman response of the breathing phonon mode ($I_{\mathrm{ph}}$) is plotted (data from ref. [27]). A coinciding dependence between $E_{G}$ and $I_{\mathrm{ph}}$ as a function of the BBO film thickness is observed. Synchrotron XRD and Raman spectroscopy experiments suggest that the strength of the oxygen-breathing mode is decreasing with thickness [25,26,27]. Therefore, we conclude that the closing of the bandgap originates from the suppression of the oxygen-breathing mode as a function of thickness.

For increasing thickness, around a thickness of 8 u.c., both the bandgap size and the intensity of the breathing phonon intensity start to increase. In both cases, the BBO films remain insulating up to the ultra-thin limit of $d_{\mathrm{BBO}}=$ 3 u.c. In addition, no step-like suppression in the breathing phonon intensity or the bandgap is observed, in good agreement with previous studies [25,26,27]. The observed c(4 × 2) surface reconstruction on the BBO films with thicknesses of 4 and 10 u.c., confirms the underlying structure is the perovskite structure and not an interfacial layer [29,37] and the identical periodicity proves that no substrate-induced strain effect is present.

The influence of the breathing phonon mode on the bandgap also explains the discrepancy between the observed bandgap of the 16-unit-cell-thick BBO film (shown in Figure 2) and the optical determined bandgap on BBO (1.2 eV versus 2.0 eV) in previous studies (for single crystals [10,11] and for films with $d_{\mathrm{BBO}}$ > 300 nm [12]). This discrepancy is a consequence of the thickness-dependent bandgap variation. In refs. [25,27], it is clearly shown that the BBO breathing phonon intensity was already affected for BBO thicknesses of 30 u.c. Therefore, the bandgap on the 16-unit-cell-thick BBO film is already reduced and not reaching the optically measured 2 eV bandgap.

## 4. Conclusions

In conclusion, by combining STM and STS, we observed a thickness-dependent wide-gap to small-gap semiconductor transition in BBO thin films. For $d_{\mathrm{BBO}}\approx$ 16 u.c., a bandgap of 1.2 ± 0.3 eV is observed. With a reduction of the film thickness, the bandgap shrinks to approximately 0.07 ± 0.04 eV for a 3-unit-cell-thick BBO film. No metallic state was detected in the ultra-thin limit. The transition is continuous and gradual and shows a coinciding dependence with the intensity of the Raman response of the breathing phonon mode as a function of thickness. A c(4 × 2) surface reconstruction is observed on the 4- and 10-unit-cell-thick BBO films, confirming the perovskite structure with the correct lattice constant underneath, excluding the influence of substrate-induced strain. The presented results show that the suppression of the oxygen-breathing mode as a function of thickness is responsible for the modification of the band gap size.

## Data Availability

Data that support the findings of this study are available from the corresponding author upon reasonable request.

## Supplementary Materials

The following are available online at https://www.mdpi.com/2079-4991/11/4/882/s1, Figure S1. (**a**) AFM image of a 4 × 4 μm area on a Nb-doped STO(001) substrate. The root-mean-square (RMS) roughness is 168 pm. The scale bar is 1 μm. (**b**) The height profile corresponding to the black line in (**a**), confirming the TiO${}_{2}$ single terminated surface. Figure S2. (**a**–**c**) RHEED images of the diffraction patterns of the 4-, 10- and 16-unit-cell-thick BBO films, respectively. The images are taken after the samples were cooled down and in high vacuum conditions. (**d**) RHEED pattern of the Nb-doped STO(001) substrate used for the 4 u.c. BBO film, taken at room temperature in high vacuum conditions. (**e**) The intensity of the main diffraction spot of the RHEED pattern is monitored during growth. The red, blue and black curves correspond to the 4-, 10- and 16-unit-cell-thick BBO films, respectively. The small black arrows indicate where the RHEED intensity was manually adjusted. Figure S3. AFM images of a 4-unit-cell-thick BBO films. The area is (**a**) 4 × 4 m, (**b**) 750 × 750 nm, (**c**) 500 × 500 nm and (**d**) 200 × 200 nm. The scale bars are equal to a quarter of the image size. The RMS value is 150 ± 20 pm. Figure S4. AFM images of (**a**,**b**) a 10 u.c. and (**c**,**d**) a 16 u.c. BBO film. The area is (**a**,**c**) 750 × 750 nm and (**b**,**d**) 200 × 200 nm. The scale bars are equal to a quarter of the image size. The RMS values for the 10- and 16-unit-cell-thick BBO films are 200 ± 20 pm and 350 ± 20 pm, respectively. Figure S5. Topography image (40 × 13 nm, scale bar 5 nm) of the 4-unit-cell-thick BBO film on Nb:STO, showing the c(4 × 2) surface reconstruction. The image is slightly distorted due to to the presence of a double tip. Inset left: Zoomed image (7.5 × 9 nm) of the c(4 × 2) reconstruction. Inset right: The corresponding Fast Fourier transform (FFT) showing the threefold symmetry with a periodicity of approximately 1 nm. The tunneling parameters are 700 pA and −1.5 V. Figure S6. d*I*/d*V* maps at different bias voltage set points. The lateral position is aligned with the topography image at the bottom (80 × 80 nm). The height difference is clearly visible in the LDOS maps for V≥0.25 V. Figure S7. (**a**–**c**) I(V) curves recorded on the 10- and 16-unit-cell-thick BBO sample. (**a**) The original I(V) curves. (**b**) The corrected I(V) curves, scaled to the set point used for the *I*(*V*) curse obtained on the 10 u.c. BBO film. (**c**) I(V) curves on semi-logarithmic scale, the gray-colored bars represent the band gap of the various measurements. (**d**–**f**) I(V) curves recorded on the 4 and 10 u.c. BBO films. (**d**) The original I(V) curves. (**e**) The corrected I(V) curves. (**f**) I(V) curves on the semi-logarithmic scale. Figure S8. Topography and spectroscopy data on 10-unit-cell-thick BBO films. (**a**) Topography of the BBO surface (200 × 200 nm, scale bar is 50 nm). (**b**) Topography image of Au(111) to show that the tip was in a good condition (100 × 100 nm, scale bar is 25 nm). (**c**) Topography scan of a 10 u.c. BBO film after scanning the Au(111) surface (20 × 20 nm, scale bar is 5 nm). The BBO surface reconstruction is visible. (**d**–**f**) The corresponding *I(V)* curves measured on the positions marked in panels (**a**–**c**), respectively. References [29,30,31,32,36,38,39] are cited in the Supplementary Materials.
