# Supplementary material for: Thickness-Dependent Band Gap Modification in BaBiO3
Source: Nanomaterials (Basel). 2021 Mar 30;11(4):882. doi: 10.3390/nano11040882 (PMC8103236; doi:10.3390/nano11040882)
Supplement: Supplementary file 1 [file nanomaterials-11-00882-s001.pdf]

Supplementary Materials: Thickness-Dependent Band Gap Modification in $BaBiO_3$

Rosa Luca Bouwmeester *, Alexander Brinkman and Kai Sotthewes

1. Sample fabrication and characterization

As a substrate Nb-doped $SrTiO_3$(001) (Nb:STO) (from CrysTec GmbH, Berlin, Germany) was used, with a doping level of 0.5 wt%. To obtain a TiO_2 single-terminated surface, a wet etching step of 30 seconds in a buffered hydrogen fluoride solution was performed [1]. Subsequently, the substrates were annealed for 1.5 hours in a furnace with a continuous oxygen flow at 930 °C. Afterwards, the surface quality was checked with an atomic force microscope (AFM). A 4 × 4 μm image of a Nb:STO is presented in Figure S1(a), nicely arranged terraces are observed. The height profile, see Figure S1(b) and corresponding to the black line in Figure S1(a), shows straight terraces with a step height of approximately 0.4 nm – in good agreement with the STO bulk lattice constant of 3.905 Å [2].

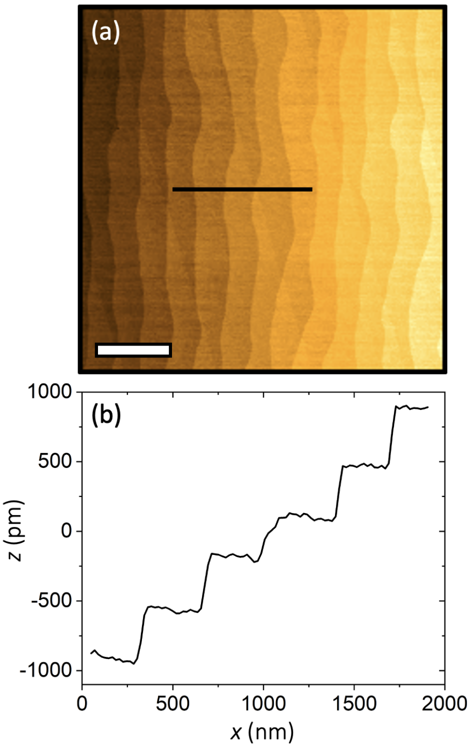

Figure S1. (**a**) AFM image of a 4 × 4 μm area on a Nb-doped STO(001) substrate. The root-mean-square (RMS) roughness is 168 pm. The scale bar is 1 μm. (**b**) The height profile corresponding to the black line in (**a**), confirming the TiO_2 single terminated surface.

The $BaBiO_3$ thin films were fabricated using pulsed laser deposition (PLD). Before each deposition, the target was sanded and a pre-ablation was performed with 600 pulses fired at 5 Hz in the same oxygen background pressure as used during the actual deposition. The same growth conditions were used as in [3]: a KrF laser at a fluence of 1.9 J/cm^2 with a repetition rate of 1 Hz, substrate temperature of 500 °C, oxygen background pressure of $1{\cdot}10^{-2}$ mbar and a substrate-target distance of 50 mm.

During the deposition, the growth of the BBO films was monitored by reflection high-energy electron diffraction (RHEED). In Figure S2(a–c) the RHEED patterns of the BBO films with thicknesses 4, 10 and 16 unit cells (u.c.), respectively, are presented. The images are taken after cool down in high vacuum conditions (average pressure of 3×10^{-7} mbar). In Figure S2(d) the diffraction pattern of the Nb:STO substrate, used for the 4-unit-cell-thick

BBO film, is shown. In Figure S2(e), the intensity of the main diffraction spot, that was monitored during growth, is presented as function of time. The red, blue and black curve correspond with the 4-, 10- and 16-unit-cell-thick BBO films, respectively. The small black arrows indicate where the intensity is manually de- or increased.

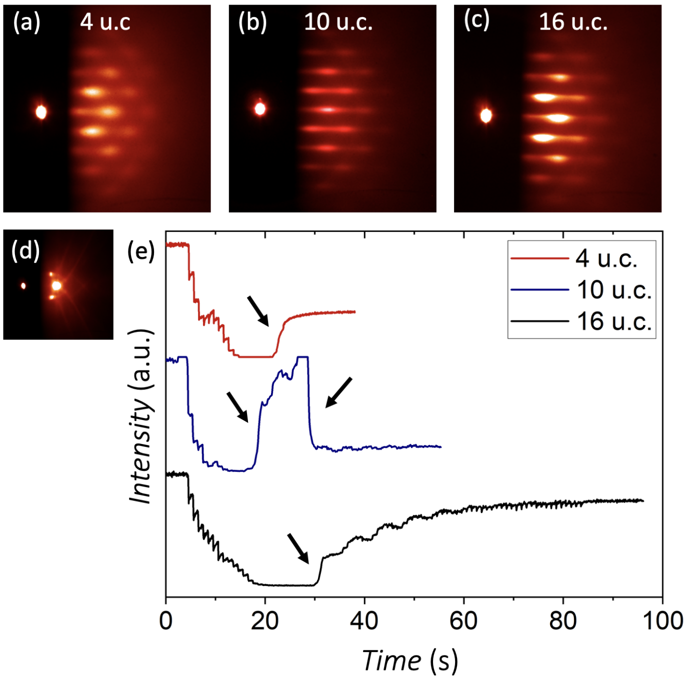

Figure S2. (**a**–**c**) RHEED images of the diffraction patterns of the 4-, 10- and 16-unit-cell-thick BBO films, respectively. The images are taken after the samples were cooled down and in high vacuum conditions. (**d**) RHEED pattern of the Nb-doped STO(001) substrate used for the 4 u.c. BBO film, taken at room temperature in high vacuum conditions. (**e**) The intensity of the main diffraction spot of the RHEED pattern is monitored during growth. The red, blue and black curves correspond to the 4-, 10- and 16-unit-cell-thick BBO films, respectively. The small black arrows indicate where the RHEED intensity was manually adjusted.

The three BBO films were in-situ transferred to a Nanoprobe scanning tunneling microscope (STM) for spectroscopy experiments. A 4-unit-cell-thick BBO film was removed from the vacuum and directly studied with an AFM, the result is presented in Figure S3. The substrate terraces are still visible, but when scanning a smaller area (the area decreases going from (a) to (d)) some roughness is observed. The root-mean-square (RMS) roughness is 150 $\pm$ 20 pm.

The same is performed for a 10- and a 16-unit-cell-thick BBO film, the AFM images are presented in Figure S4(a, b) and (c, d), respectively. The average RMS roughnesses are 200 $\pm$ 20 pm and 350 $\pm$ 20 pm for the 10 and 16 u.c. BBO films, respectively. The RMS values are used as a measure for the x-axis error bars in Figure 5 of the main text.

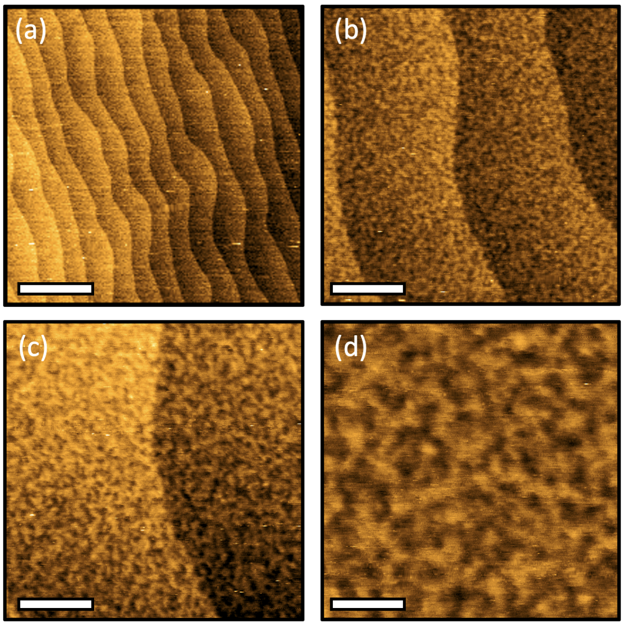

Figure S3. AFM images of a 4-unit-cell-thick BBO films. The area is (**a**) 4 × 4 μm, (**b**) 750 × 750 nm, (**c**) 500 × 500 nm and (**d**) 200 × 200 nm. The scale bars are equal to a quarter of the image size. The RMS value is 150 ± 20 pm.

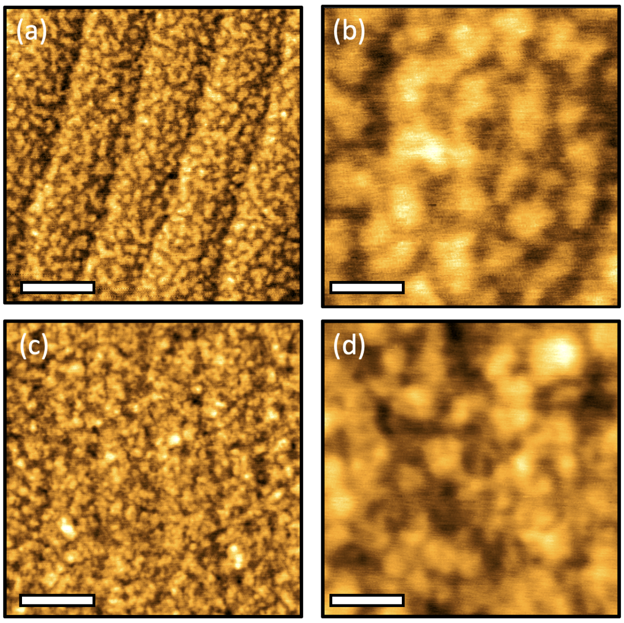

Figure S4. AFM images of (**a**,**b**) a 10 u.c. and (**c**,**d**) a 16 u.c. BBO film. The area is (**a**,**c**) 750 × 750 nm and (**b**,**d**) 200 × 200 nm. The scale bars are equal to a quarter of the image size. The RMS values for the 10- and 16-unit-cell-thick BBO films are 200 ± 20 pm and 350 ± 20 pm, respectively.

2. Scanning Tunneling Microscopy

The results of the spectroscopy experiments with the 4-, 10- and 16-unit-cell-thick BBO films are presented in the main text. Below, some additional results are presented.

2.1. Surface Reconstruction of a 4-Unit-Cell-Thick Film

Figure S5 shows the surface of the 4-unit-cell-thick BBO film on Nb:STO. The image is slightly distorted due the presence of a double tip. The atoms are arranged in the same

symmetry and periodicity as for the 10-unit-cell-thick BBO films (Figure 1 of the main text), corresponding to a c(4 × 2) surface reconstruction.

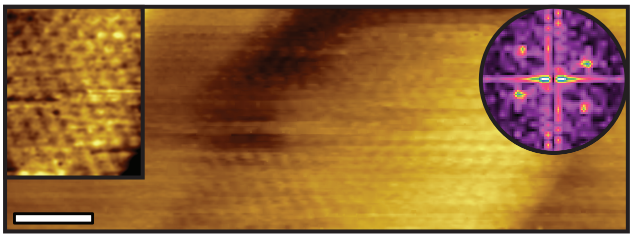

Figure S5. Topography image (40 × 13 nm, scale bar 5 nm) of the 4-unit-cell-thick BBO film on Nb:STO, showing the c(4 × 2) surface reconstruction. The image is slightly distorted due to to the presence of a double tip. Inset left: Zoomed image (7.5 × 9 nm) of the c(4 × 2) reconstruction. Inset right: The corresponding Fast Fourier transform (FFT) showing the threefold symmetry with a periodicity of approximately 1 nm. The tunneling parameters are 700 pA and -1.5 V.

2.2. LDOS 4 u.c. BBO film

In Figure S6 the spatially resolved local density of states (LDOS) for the 4-unit-cell-thick BBO film is shown for different bias voltages. Around the Fermi energy, no contrast is observed, similar as for the 10-unit-cell-thick BBO film (see Figure 3(d) in the main text). Only for $V > 0$ V, clear correlations are observed between the LDOS maps and the topography (bottom most image, same as inset of Figure 4(a) in the main text). The higher region (see red dot in the inset of Figure 4(a) in the main text) has a significantly lower LDOS than the lower region (blue dot).

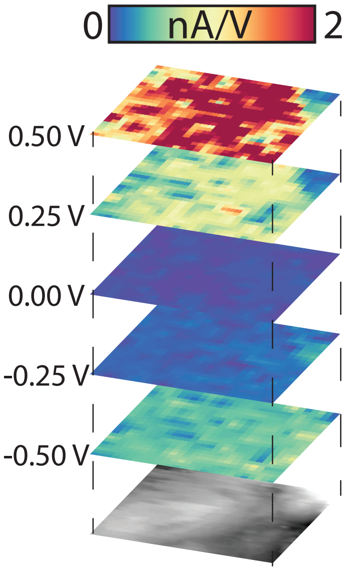

Figure S6. $\mathrm{d}I/\mathrm{d}V$ maps at different bias voltage set points. The lateral position is aligned with the topography image at the bottom (80 × 80 nm). The height difference is clearly visible in the LDOS maps for $V \geq 0.25$ V.

2.3. Band Gap Determination

In order to properly compare the band gap measured on the different samples, the $I(V)$ curves are first scaled to the same set point. In Figure S7(a–c) the 10- and 16-unit-cell-thick BBO films are compared. Since it was not possible to use the same scan settings on both surfaces, the $I(V)$ spectrum of the 16 u.c. BBO film is scaled to the set point used for the $I(V)$ curves obtained on the 10 u.c. BBO film (I = 600 pA and $V = -1.5$ V, Figure S7(b)). The size of the band gap (E_G) is determined for all measurements by plotting the corrected

$I(V)$ spectra on a semi-logarithmic scale (see Figure S7(c)) and, subsequently, taking the average voltage separation between the conduction band and valence band current onsets at the lowest detectable current (detection limit approximately 500 fA) [4–6].

From this measure a difference in the band gap is obtained between the 10 and 16 u.c. BBO films (shown in Figure S7(c)). A similar approach is used to compare the 4 and 10 u.c. BBO samples, presented in Figure S7(d-f). The obtained $I(V)$ curve on the 10-unit-cell-thick BBO film is scaled with respect to the $I(V)$ curve measure on the 4-unit-cell thick BBO film (I = 400 pA and V = 1 V). Although the absolute value for the band gap extracted from STS spectra depends slightly on the chosen scan parameters, the increasing trend between the thickness and the size of the band gap (Figure 5 in the main text) remains unaffected.

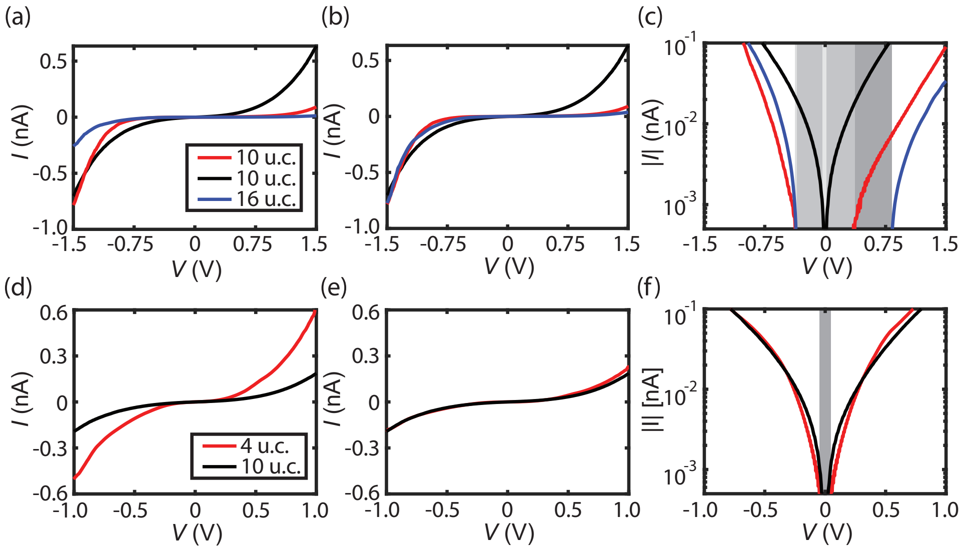

Figure S7. (**a–c**) $I(V)$ curves recorded on the 10- and 16-unit-cell-thick BBO sample. (**a**) The original $I(V)$ curves. (**b**) The corrected $I(V)$ curves, scaled to the set point used for the I(V) curse obtained on the 10 u.c. BBO film. (**c**) $I(V)$ curves on semi-logarithmic scale, the gray-colored bars represent the band gap of the various measurements. (**d–f**) $I(V)$ curves recorded on the 4 and 10 u.c. BBO films. (**d**) The original $I(V)$ curves. (**e**) The corrected $I(V)$ curves. (**f**) $I(V)$ curves on the semi-logarithmic scale.

2.4. Topography and Spectroscopy

Some of the topography images look a little bit scratchy, even though the measurements are repeatedly performed with different tips and scanners (the measurements are performed in the Nanoprobe STM which contains four indepently operating scanners). In order to exclude the possibility that the tips are contaminated, topography and spectroscopy data is presented in Figure S8, taken before and after scanning on Au(111).

In Figure S8(a), a topography scan on a 10-unit-cell-thick BBO sample is presented. The topography looks a bit scratchy, but terraces are still visible. The measured $I(V)$ curve is displayed in Figure S8(d) and shows the presence of a band gap. After the measurement on the BBO film, the sample was replaced by Au(111) (see Figure S8(b)). Several steps are visible and on the terrace the herringbone reconstruction is present [7], indicating that the quality of the tip is good. Also, a metallic spectrum is measured in the $I(V)$ measurement, presented in Figure S8(e).

Subsequently, the 10-unit-cell-thick BBO film is scanned. A higher quality topography scan is obtained, see Figure S8(c), with a similar spectroscopy measurement (Figure S8(f)) as initially observed on BBO (Figure S8(d)). Although the same tip and tunneling parameters are used, the image quality is slightly improved implicating that the scratchy topography appearance is not caused by the tip quality but reflects the state of the surface. Furthermore, the topography image of Figure S8(a) (and also of Figure 3 and 4 of the main text) look very similar to the topography images scanned with the AFM in Figure S3 and Figure S4, suggesting that it is a not a tip artifact that is measured.

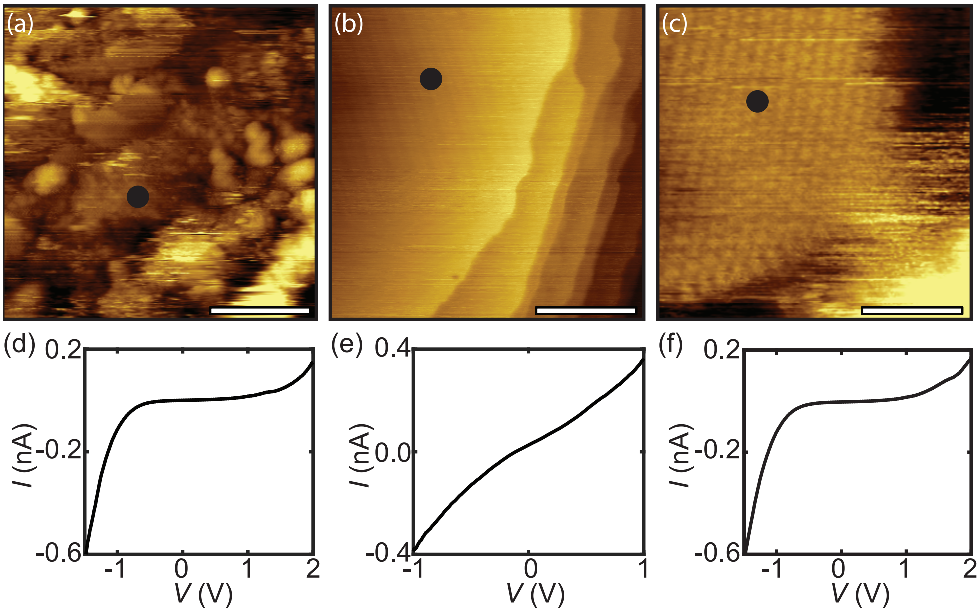

Figure S8. Topography and spectroscopy data on 10-unit-cell-thick BBO films. (**a**) Topography of the BBO surface (200 × 200 nm, scale bar is 50 nm). (**b**) Topography image of Au(111) to show that the tip was in a good condition (100 × 100 nm, scale bar is 25 nm). (**c**) Topography scan of a 10 u.c. BBO film after scanning the Au(111) surface (20 × 20 nm, scale bar is 5 nm). The BBO surface reconstruction is visible. (**d**–**f**) The corresponding *I*(*V*) curves measured on the positions marked in panels (**a**–**c**), respectively.

References

1. Koster, G.; Kropman, B.L.; Rijnders, G.J.H.M.; Blank, D.H.A.; Rogalla, H. Quasi-ideal strontium titanate crystal surfaces through formation of strontium hydroxide. *Applied Physics Letters* **1998**, *73*, 2920–2922. doi:10.1063/1.122630.
2. Ohtomo, A.; Hwang, H.Y. A high-mobility electron gas at the $LaTiO_3/SrTiO_3$ heterointerface. *Nature* **2004**, *427*, 423–426.
3. Bouwmeester, R.L.; de Hond, K.; Gauquelin, N.; Verbeeck, J.; Koster, G.; Brinkman, A. Stabilization of the perovskite phase in the Y–Bi–O system by using a $BaBiO_3$ buffer layer. *Phys. Status Solidi RRL* **2019**, *13*, 1800679.
4. Feenstra, R.; Stroscio, J. Tunneling spectroscopy of the GaAs(110) surface. *J. Vac. Sci. Technol. B* **1987**, *5*, 923.
5. Ebert, P.; Schaafhausen, S.; Lenz, A.; Sabitova, A.; Ivanova, L.; Dahne, M.; Hong, Y.L.; Gwo, S.; Eisele, H. Direct measurement of the band gap and Fermi level position at InN(1120). *Appl. Phys. Lett.* **2011**, *98*.
6. Herbert, F.W.; Krishnamoorthy, A.; van Vliet, K.J.; Yildiz, B. Quantification of electronic band gap and surface states on FeS_2(100). *Surf. Sci.* **2013**, *618*, 53–61.
7. Wöll, C.; Chiang, S.; Wilson, R.; P.H., L. Determination of Atom Positions at Stacking-Fault Dislocations on Au(111). *Phys. Rev. B* **1989**, *39*, 7988.
